# Screening-positive attention-deficit/hyperactivity disorder symptoms among incarcerated individuals in Paraguay: prevalence, psychological correlates, and criminological context

**DOI:** 10.3389/fpsyt.2026.1752901

**Published:** 2026-04-15

**Authors:** Julio Torales, Lia Gonçalves Possuelo, Iván Barrios, Gladys Estigarribia, Gisselle Velázquez, Marcelo O’Higgins, João Mauricio Castaldelli-Maia, Antonio Ventriglio, Alexander Smith, Ana Buadze, Michael Liebrenz

**Affiliations:** 1 Universidade de Santa Cruz do Sul, Santa Cruz do Sul, RS, Brazil; 2 Grupo de Investigación sobre Epidemiología de los Trastornos Mentales, Psicopatología y Neurociencias, Facultad de Ciencias Médicas, Universidad Nacional de Asunción, San Lorenzo, Paraguay; 3 Vicerrectoría de Investigación y Postgrado, Universidad de Los Lagos, Osorno, Chile; 4 Facultad de Ciencias de la Salud, Universidad Sudamericana, Pedro Juan Caballero, Paraguay; 5 Cátedra de Bioestadística, Facultad de Ciencias Médicas, Universidad Nacional de Asunción, Santa Rosa del Aguaray, Paraguay; 6 Instituto Regional de Investigación en Salud, Universidad Nacional de Caaguazú, Coronel Oviedo, Paraguay; 7 Department of Psychiatry, University of São Paulo, São Paulo, SP, Brazil; 8 Department of Clinical and Experimental Medicine, University of Foggia, Foggia, Italy; 9 Department of Forensic Psychiatry, University of Bern, Bern, Switzerland; 10 Department of Psychiatry, Psychotherapy and Psychosomatics, Psychiatric Hospital, University of Zurich, Zurich, Switzerland

**Keywords:** ADHD symptoms, emotional dysregulation, incarceration, Paraguay, suicidality

## Abstract

**Background:**

Attention-Deficit/Hyperactivity Disorder (ADHD) symptoms are frequently reported among incarcerated populations and have been associated with impulsivity, emotional dysregulation, and adverse mental health outcomes. Despite growing international evidence, no previous studies have examined screening-positive ADHD symptoms among prisoners in Paraguay or evaluated Spanish-language screening tools in correctional settings.

**Aim:**

To estimate the prevalence of screening-positive ADHD symptoms among incarcerated men and women in Paraguay, examine selected psychological and criminological variables, and assess the reliability and factorial structure of the Spanish version of the Symptom Check List–ADHD (SCL-ADHD).

**Methods:**

This cross-sectional study included 836 inmates (621 men, 215 women) recruited through probabilistic sampling in three Paraguayan prisons. Screening-positive ADHD status was defined as a score ≥ 12 on the nine-item SCL-ADHD derived from the SCL-90-R. Psychiatric symptoms, suicide risk, and substance-related problems were assessed using validated measures. Multivariable logistic regression identified independent correlates of screening-positive ADHD status.

**Results:**

Screening-positive ADHD status was observed in 33.4% of participants, with higher prevalence among women than men (39.1% vs. 31.4%; OR = 1.40, 95% CI 1.02–1.93, p = 0.04). Screening-positive ADHD was independently associated with suicide risk (OR = 3.85, p < 0.001) and elevated SCL-90-R dimensions of hostility, anxiety, depression, and obsessive–compulsive symptoms. Sensitivity analyses using continuous symptom scores showed associations with hostility, anxiety, and obsessive–compulsive symptoms remained, whereas association with depression attenuated. No significant independent associations were observed with criminological variables. The Spanish SCL-ADHD demonstrated acceptable internal consistency (α = 0.76) and a coherent symptom structure.

**Conclusion:**

Screening-positive ADHD symptoms were common among incarcerated individuals in Paraguay, particularly women, and were associated with concurrent emotional dysregulation and suicidality. These findings reflect screening-based symptom burden rather than confirmed adult ADHD diagnoses and highlight the potential utility of systematic ADHD screening within correctional mental health services.

## Introduction

Mental disorders are highly prevalent among incarcerated populations and represent a major global public health issue. The World Health Organization (WHO) has affirmed that prisons are environments of extreme psychosocial stress, where overcrowding, isolation, violence, and poor access to healthcare amplify psychiatric morbidity (1). Systematic reviews estimate that rates of severe mental illness among prisoners are several times higher than in the general population (2, 3). Recent evidence indicates that comparable trends persist in carceral settings globally and that untreated psychiatric conditions are linked to suicide, self-harm, and elevated post-release mortality (4, 5). In Latin America, where forensic psychiatry services are scarce, the mental health gap is particularly pronounced (6, 7).

Paraguay exemplifies these challenges. With one of the highest incarceration rates in the region and chronic prison overcrowding (often exceeding 350% capacity), the Paraguayan correctional system faces structural limitations in mental-health service provision (8). Despite these conditions, empirical studies of mental disorders among incarcerated people in Paraguay remain limited, and no data exist regarding neurodevelopmental disorders, such as attention-deficit/hyperactivity disorder (ADHD). This knowledge gap impedes the design of evidence-based interventions for potentially highly vulnerable populations.

ADHD is a neurodevelopmental condition characterized by inattention, hyperactivity, and impulsivity, with persistence into adulthood in up to 60% of cases (9, 10). Adult ADHD is associated with emotional dysregulation, substance use, and elevated risks of impulsive or violent behaviour (11, 12). Meta-analyses suggest that the prevalence of ADHD among incarcerated adults ranges from 20% to 30%, which is five to ten times higher than in the community (13, 14).

Beyond prevalence, identifying ADHD in correctional settings has important clinical, institutional, and public safety implications. Inmates with ADHD show higher rates of behavioural disturbances, disciplinary infractions, and violent incidents compared with non-ADHD inmates, posing significant challenges for prison management (15–17). Longitudinal and meta-analytic evidence further indicates that individuals with ADHD reoffend more and have significantly higher recidivism rates, even after controlling for antisocial personality disorder and other criminogenic factors (18–21). In addition, incarcerated individuals with ADHD are a particularly vulnerable subgroup, characterized by higher psychiatric comorbidity, greater psychosocial adversity, and increased risk of victimization and interpersonal violence, including intimate partner violence (22–25).

From a mechanistic perspective, ADHD-related impairments in executive functioning, impulse control, and emotional regulation are thought to mediate the association between ADHD symptoms and criminal behaviour. Inhibitory deficits, heightened stress reactivity, and difficulties in planning and delay of gratification may increase susceptibility to aggressive responses, substance misuse, and rule violations, particularly within the highly stressful and restrictive prison environment (26, 27). Importantly, while incarceration itself may induce hyperarousal, irritability, and attentional difficulties, ADHD is conceptualized as a neurodevelopmental condition with symptom onset prior to imprisonment and relative persistence across contexts, underscoring the need for careful clinical differentiation rather than diagnostic substitution.

Sex differences in ADHD among incarcerated populations warrant further attention. Although ADHD is historically underdiagnosed in females, emerging evidence indicates that women in correctional settings may exhibit equal or higher rates of ADHD symptoms than men, often presenting with less overt hyperactivity and more internalising or emotionally dysregulated profiles (28, 29). These patterns could have sizable implications for detection and treatment, particularly given the elevated burden of trauma, self-harm, and comorbid mood disorders observed among incarcerated women.

Despite growing international awareness, ADHD remains largely undiagnosed and untreated in correctional settings, especially in low- and middle-income countries. Timely identification is critical, as evidence-based interventions have been shown to improve behavioural regulation and may attenuate institutional misconduct and long-term recidivism. Pharmacological treatments, particularly stimulant medications, have demonstrated efficacy in ameliorating ADHD symptoms and behavioural problems among incarcerated adults (30–32). Equally, emerging psychotherapeutic approaches, including cognitive-behavioural interventions adapted for prison settings, show promising results (33, 34). However, the implementation of comprehensive diagnostic assessments in overcrowded and resource-limited prison systems remains challenging.

In this context, brief screening instruments represent a pragmatic first step for identifying individuals who may benefit from further assessment and intervention. The Symptom Checklist-ADHD (SCL-ADHD), a nine-item subscale derived from the widely used Symptom Checklist-90-Revised (SCL-90-R), offers several advantages for correctional settings: it is brief, requires no specialised training, can be administered alongside routine mental health assessments, and has demonstrated acceptable sensitivity for detecting probable adult ADHD (9). Nevertheless, its performance has not previously been evaluated in Latin American or Spanish-speaking prison populations.

The assessment of ADHD in correctional settings entails important diagnostic challenges. Core ADHD symptoms—such as inattention, impulsivity, and emotional dysregulation—overlap with manifestations of trauma-related disorders, substance use disorders, mood disorders, and personality pathology. Prison populations present disproportionately high rates of post-traumatic stress disorder (PTSD), substance dependence, and affective instability (35, 36), and adult ADHD itself is characterized by substantial psychiatric comorbidity (37, 38). Consequently, elevated ADHD symptom scores in custodial contexts may reflect neurodevelopmental vulnerability, incarceration-related distress, comorbid psychopathology, or their interaction, requiring cautious differential interpretation (39).

Beyond epidemiological estimation, systematic screening for ADHD symptoms in correctional settings has tangible clinical and operational implications. Early identification of inmates with elevated ADHD symptom burden may facilitate targeted diagnostic evaluation, individualized treatment planning, and appropriate allocation of mental health resources. From an institutional perspective, recognizing neurodevelopmental vulnerabilities may improve risk management strategies, reduce behavioural incidents, and enhance responsivity to rehabilitative programs. Furthermore, timely screening may contribute to continuity of care during the transition from incarceration to community reintegration, where untreated ADHD is associated with substance relapse, impulsive behaviour, and recidivism (12, 18). In resource-limited prison systems, brief screening tools may therefore represent a pragmatic entry point for structured mental health intervention pathways.

Accordingly, the present study had the following objectives: (1) to estimate the prevalence of screening-positive ADHD status among incarcerated adults in Paraguay; (2) to examine the psychometric performance of the Spanish version of the SCL-ADHD in this context; and (3) to explore psychological, clinical, and selected criminological variables in relation to screening-positive ADHD symptoms, including psychiatric comorbidity, suicidality, substance use, and offence-related characteristics.

Consequently, this research contributes to addressing the empirical gap regarding screening-positive ADHD symptoms in correctional settings in Latin America, providing both epidemiological and psychometric evidence. It further offers a pragmatic framework for integrating brief ADHD screening into routine prison mental health assessment, thereby informing service planning and policy development in Paraguay and comparable resource-limited contexts.

## Methods

### Design and setting

This descriptive, analytical, cross-sectional study based on secondary data analysis was carried out as part of the national research project PINV01-899: *Clinical and Epidemiological Analysis of the Mental Health of People Deprived of Liberty in Paraguay*. Three major penitentiary institutions located in different regions of the country participated, representing both male and female populations under the jurisdiction of the Paraguayan Ministry of Justice. Data collection was conducted between October 2024 and March 2025.

The cross-sectional design enabled estimation of the prevalence of screening-positive ADHD status at a specific point in time. The study was based on secondary data analysis, as screening-positive ADHD classification was derived *post hoc* from previously administered items of the SCL-90-R, rather than from assessment of past exposures or outcomes. This methodological strategy, successfully employed by Eich et al. (9) for developing the SCL-ADHD subscale, allows new analyses to be performed on existing data while maintaining psychometric integrity. Although causal inference is not possible within this design, it is appropriate for identifying clinical and criminological correlates and generating hypotheses for future prospective studies. Reporting followed the STROBE guidelines for observational research (40).

### Participants and sampling

The target population comprised adults (≥ 18 years) incarcerated in the national prison system. A simple random probabilistic sampling method was implemented independently in each institution to ensure equal probability of selection. At each participating institution, the sampling frame was constructed using updated administrative prison registries that included all eligible inmates at the time of data collection. Simple random selection was performed using a computerized random number generator. The randomization procedure was conducted by trained members of the research team who were not involved in clinical assessment or data analysis, thereby minimizing selection bias and ensuring allocation concealment at the sampling stage.

The final sample included 836 participants (621 men and 215 women), constituting the largest consolidated national dataset on mental health in Paraguayan prisons to date. The required sample size was estimated using Epidat 4.2 (Pan American Health Organization, Health Board of Galicia, and Universidad CES de Colombia), assuming a 26.2% expected ADHD prevalence (14), a 95% confidence level, and a 3,5% precision. The theoretical minimum of 607 participants was therefore exceeded, ensuring adequate statistical power for multivariable analyses (41).

Inclusion criteria involved participants being 18 years or older and having complete responses to all nine items comprising the SCL-ADHD subscale. Participants were excluded if they had missing data in two or more of those items or if response patterns were clearly invalid (e.g., identical scores across the entire questionnaire). No participant was removed due to comprehension difficulties or language barriers, and none withdrew from participation once enrolled. Since this study was conceived as a screening-based epidemiological investigation and represents a secondary analysis of data collected for a broader mental health survey, prior clinical diagnosis of ADHD, age at diagnosis, symptom onset, or developmental course were not stipulated as inclusion criteria. The objective was to identify screening-positive ADHD symptomatology at the time of assessment rather than to reconstruct longitudinal diagnostic trajectories.

### Measures

#### Sociodemographic and prison-context variables

Information was collected on age, nationality (Paraguayan or foreign), Indigenous status (yes/no), educational level (none, primary, secondary, tertiary), prior employment (yes/no), monthly income relative to the national minimum wage (below, equal to, above), and parental status. Prison-related variables included legal situation (sentenced > 5 years, sentenced ≤ 5 years, or remanded), with the 5-year threshold reflecting a legal distinction in the Paraguayan criminal justice system between less severe and more serious offences, which entails differences in sentencing severity, custodial conditions, and penitentiary regimes; principal offence type (violent, drug-related and sexual offences), confinement in a maximum-security regime (yes/no), prior incarcerations (yes/no), age at first imprisonment, and duration of current imprisonment (in years).

#### Health-related variables

Participants indicated whether they had any medical conditions or previous psychiatric diagnoses and whether they were currently receiving treatment, specifying the type (medical, psychological, or psychiatric). However, specific information regarding prior ADHD diagnosis, age at ADHD diagnosis, lifetime treatment with stimulant or non-stimulant medication, or longitudinal psychiatric history was not systematically collected in the parent dataset and was therefore not available for the present secondary analysis. Medication status at the time of assessment was recorded in general terms (i.e., whether treatment was being received), but detailed pharmacological classification was not available.

#### Symptom checklist-90-revised

The *SCL-90-R* (42, 43) is a 90-item self-report instrument evaluating psychological distress across nine symptom dimensions (Somatization, Obsessive-Compulsive, Interpersonal Sensitivity, Depression, Anxiety, Hostility, Phobic Anxiety, Paranoid Ideation, and Psychoticism). It also encompasses three global indices: the Global Severity Index (GSI), Positive Symptom Total (PST), and Positive Symptom Distress Index (PSDI). Each item is rated on a five-point Likert scale from 0 (“not at all”) to 4 (“extremely”). The SCL-90-R provides raw scores that can be transformed into standardized T-scores to facilitate clinical interpretation. According to the original manual, “caseness” is typically defined by a T-score ≥ 63 on at least one primary symptom dimension or by T-scores ≥ 63 on two or more global indices (GSI, PST, PSDI) (42). In the present study, symptom dimensions were categorized into “at-risk” groups informed by this established clinical framework to facilitate interpretation of clinically meaningful symptom burden without implying formal diagnostic caseness. For multivariable modelling, SCL-90-R symptom dimensions were primarily entered as dichotomous variables (“at-risk” vs. not at-risk) based on the conventional T-score ≥ 63 threshold. This operationalization was selected to enhance clinical interpretability and comparability with established caseness criteria, while recognizing the potential loss of information inherent to dichotomization. As a sensitivity analysis, the logistic regression model was re-estimated using continuous T-scores for the same symptom dimensions in order to evaluate the robustness of associations and to reduce the risk of artificial inflation of effect sizes due to dichotomization.

The Spanish adaptation by Casullo and Pérez (44) for the Universidad de Buenos Aires and CONICET was employed. This version has demonstrated robust reliability and validity across South American samples (45–47) and requires about 15 minutes to complete. Internal consistency was evaluated using Cronbach’s α, which showed excellent reliability in the present sample.

In this investigation, SCL-90-R scores reflect current psychological distress as reported at the time of assessment and do not allow differentiation between pre-incarceration symptomatology and distress potentially exacerbated by the prison environment. As a self-report measure administered in a high-stress custodial context, responses may be influenced by situational factors; therefore, SCL-90-R dimensions were interpreted as indicators of symptom burden and not as evidence of formal psychiatric diagnoses or as substitutes for structured diagnostic assessments.

#### SCL-ADHD subscale

Screening-positive ADHD status was assessed using the SCL-ADHD, a nine-item subset of the SCL-90-R developed by Eich et al. (9) to screen for adult ADHD symptoms of inattention, hyperactivity, and impulsivity. In the original study, exploratory factor analysis identified a three-factor solution labeled “Nervousness,” “Cognitive Impairment,” and “Irritability,” reflecting distinct but correlated symptom clusters within the ADHD construct (9). This empirical structure informed the specification of the confirmatory model tested in the present study. Total scores range from 0 to 36, with higher values indicating greater symptom severity. Following the recommendation of the original validation study, a cut-off score of ≥ 12 was applied to classify screening-positive ADHD status. This threshold has shown satisfactory sensitivity (75%) and specificity (54%) for identifying likely cases in adult populations (9). Although the original validation study was conducted in a clinical outpatient sample, the recommended cut-off was retained for comparability. In correctional contexts characterized by high psychiatric comorbidity, screening classifications should be interpreted cautiously as indicators of elevated symptom burden rather than confirmed diagnoses.

This investigation represents the first application of the SCL-ADHD derived from the validated Spanish SCL-90-R in a Latin American correctional sample. All SCL-ADHD items were drawn verbatim from the previously validated Spanish version of the SCL-90-R (45–47); therefore, no *de novo* translation or linguistic validation was performed for the present study. Nevertheless, to ensure transparency and contextual appropriateness, a semantic equivalence review was undertaken, comparing the original English and German item formulations reported by Eich et al. (9) with the validated Spanish SCL-90-R wording. Minor lexical clarifications were introduced solely to optimize clarity for Paraguayan Spanish speakers while preserving the original meaning of the items. These clarifications do not constitute a formal translation or re-translation process. The documentation of semantic equivalence is provided in **Appendix I**, and the final Spanish wording used in this study is presented in **Appendix II**. Because the SCL-ADHD is not an independent instrument but a subscale derived directly from the SCL-90-R, and because all items were taken from a previously validated Spanish adaptation of the SCL-90-R with established psychometric properties in South American populations, a full forward–backward translation procedure was not performed. The present procedure therefore represents contextual refinement of an already validated Spanish instrument rather than a new linguistic validation process. Nevertheless, the absence of formal back-translation is acknowledged as a methodological limitation, and findings should be interpreted accordingly.

In this investigation, SCL-ADHD scores reflect current symptom burden as reported at the time of assessment, without distinction between pre-incarceration and incarceration-related symptom onset. Duration of current imprisonment and prior incarcerations were collected separately and analysed as contextual variables. As with all screening instruments, the SCL-ADHD is intended to maximize sensitivity rather than diagnostic specificity. The use of a validated cut-off and the examination of convergent patterns with related psychopathology aimed to reduce misclassification; however, false positives and false negatives cannot be fully excluded in the absence of gold-standard diagnostic interviews. Accordingly, classification as screening-positive does not constitute a clinical diagnosis of ADHD, which would require evidence of developmental onset, cross-context persistence, and structured clinical assessment.

Internal consistency was evaluated using Cronbach’s α, and a confirmatory factor analysis (CFA) using maximum-likelihood estimation tested the model proposed by the authors. Model-fit indices (χ^2^/df, CFI, TLI, RMSEA, and SRMR) were interpreted according to the criteria proposed by Schermelleh-Engel, Moosbrugger, and Müller (48).

#### Columbia-suicide severity rating scale

Suicidal ideation and behaviour were measured using the six-item *C-SSRS Screener* (49), which includes five items on suicidal thoughts and one on suicidal behaviour. The officially authorized Spanish version from the Columbia Lighthouse Project was used. The scale has demonstrated good predictive validity and reliability across diverse clinical and institutional contexts (50, 51). The screener captures the presence of suicidal ideation and behaviour at the time of assessment and does not reconstruct lifetime trajectories or prior clinical diagnoses.

#### MULTICAGE CAD-4

Substance-related and behavioural addictions were evaluated using the *MULTICAGE CAD-4* (52), a 32-item questionnaire originally developed in Spanish that explores eight domains of addictive behaviour following the CAGE structure (53). Each domain comprises four dichotomous items addressing perception of control, external concern, guilt, and withdrawal. Two or more affirmative responses per subscale indicate probable problematic behaviour. For this study, only the alcohol and illicit-substance subscales were applied, given their particular relevance to incarcerated populations. Prior research has supported the reliability and construct validity of this instrument in Paraguayan samples (54). Responses reflect self-reported indicators of problematic use at the time of evaluation rather than formal substance use disorder diagnoses.

Both the C-SSRS Screener and the MULTICAGE CAD-4 consist of dichotomous (yes/no) items and are designed as screening instruments rather than internally consistent psychometric scales. Accordingly, internal consistency indices such as Cronbach’s α were not calculated, as these measures are intended to identify the presence of clinically relevant indicators rather than to measure a single latent construct.

### Procedure and ethical considerations

All interviews were conducted face-to-face in secure areas of each prison. The research team consisted of psychiatrists and psychologists with postgraduate qualifications and specific training in all instruments. Prior to data collection, all assessors completed a structured training session that included standardised administration protocols, role-play exercises, and calibration discussions to ensure procedural consistency across institutions. Initial interviews were supervised by the principal investigator (J.T.), and periodic random observations were performed to ensure protocol adherence. These observations consisted of unannounced supervisory visits during data collection sessions, focusing on correct instrument administration, adherence to standardized interview procedures, and compliance with ethical requirements. Protocol adherence was monitored qualitatively through direct observation and feedback, rather than through formal quantitative fidelity scoring. Any procedural discrepancies identified during supervisory visits were discussed in team meetings and resolved through consensus to maintain uniform application of the assessment protocol.

Participants received full information regarding study aims, confidentiality, and voluntary participation. Written informed consent was obtained from every participant. Educational attainment was recorded in seven categories (primary complete/incomplete; secondary complete/incomplete; no formal education; university complete/incomplete). To ensure adequate comprehension across literacy levels, all instruments were administered by trained clinicians in an interviewer-assisted format; when necessary, items were read aloud verbatim following standardized instructions. No participant was excluded due to literacy or language barriers. Each assessment lasted approximately 55–75 minutes and was conducted individually or in pairs, according to institutional safety recommendations. Participants exhibiting acute distress were referred immediately to prison health services.

This study was conducted within the framework of the Scientific Research Improvement Program of the Research Group on Epidemiology of Mental Disorders, Psychopathology, and Neurosciences and received approval from the Department of Medical Psychology of the School of Medical Sciences, National University of Asunción, Paraguay (Reference 001-010-2025). All data were anonymized, stored in password-protected files, and handled in accordance with ethical standards for research involving human subjects.

### Data analysis

Databases from the three participating correctional institutions were merged into a single anonymized dataset. A comprehensive codebook was developed specifying the operational definitions of each variable, the criteria for derived variables (e.g., age categories and clinical indices), and the procedures for managing missing values. Data cleaning included range verification, detection of outliers, identification of duplicate cases, and logical consistency checks, such as verifying chronological coherence between current age and age at first incarceration. To ensure integrity, 10% of all cases were manually audited and cross-verified against the original records, following recommendations for reproducible research in mental health epidemiology (55).

Patterns of missing data were examined using Little’s MCAR test to determine whether the data were Missing Completely at Random (56). When less than 10% of observations were missing for a given variable, missing values were handled through multiple imputation by chained equations, following Rubin’s (57) framework and van Buuren’s (58) methodological updates. Analyses were performed using *Stata 18* (StataCorp, College Station, TX, USA) and *R 4.3* (R Foundation for Statistical Computing, Vienna, Austria), with version-controlled scripts to guarantee analytical reproducibility.

Continuous variables were summarized as means with standard deviations and interquartile ranges, whereas categorical variables were expressed as absolute and relative frequencies. The prevalence of screening-positive ADHD status, defined as a score ≥ 12 on the SCL-ADHD, was estimated with 95% confidence intervals. Bivariate associations between screening-positive ADHD status and categorical predictors were evaluated using Pearson’s chi-square test, while comparisons of continuous variables between groups were made using independent-samples *t* tests or Mann-Whitney U tests when the assumption of normality was not met (59).

Subsequently, multivariable logistic regression models were fitted to identify independent sociodemographic, clinical, and criminological correlates of screening-positive ADHD status. Given that this study constitutes a secondary analysis of data originally collected for a broader national mental health survey, the multivariable modelling strategy was conceived as exploratory and hypothesis-generating rather than confirmatory. Variable availability was determined by the original survey design, and no *a priori* model specific to ADHD had been pre-specified at the time of data collection. Predictor selection followed a structured two-stage exploratory approach. In the first stage, bivariate analyses were conducted to identify a restricted set of candidate covariates, primarily to reduce model overparameterization and preserve statistical stability given the number of outcome events. In the second stage, candidate predictors were entered jointly into a multivariable binary logistic regression model and retained only if they remained statistically significant (p < 0.05) and contributed meaningfully to overall model fit, as evaluated by likelihood-ratio tests and the Akaike Information Criterion (AIC). Bivariate analyses were employed as an initial screening step; however, variables were not entered automatically into the multivariable model solely on the basis of bivariate statistical significance. Instead, candidate predictors were evaluated jointly, and variables that independently contributed to overall model fit, as assessed by the omnibus likelihood-ratio test, were retained in the final model. This strategy allowed exclusion of variables whose effects were attenuated or redundant in the multivariable context, while reducing collinearity and overfitting. Model adequacy was examined using omnibus likelihood-ratio tests, and results were expressed as adjusted odds ratios (aOR) with 95% confidence intervals (60). Multicollinearity was assessed using variance inflation factors (VIF), with values below conventional thresholds indicating acceptable model stability. Prison-context variables (legal situation, duration of current imprisonment, prior incarcerations, age at first imprisonment, and maximum-security regime) were initially examined in bivariate analyses and subsequently evaluated within the multivariable modelling framework. Variables that did not independently contribute to overall model fit were not retained in the final model.

To evaluate the robustness of the findings and address potential concerns regarding dichotomization of symptom dimensions and shared method variance, a sensitivity analysis was conducted. The logistic regression model was re-estimated entering SCL-90-R symptom dimensions as continuous T-scores rather than dichotomized “at-risk” variables. This alternative specification allowed examination of the stability of effect estimates and reduction of potential inflation of odds ratios associated with threshold-based categorization. To assess potential construct overlap among symptom predictors derived from the same assessment framework, Pearson correlation coefficients were estimated between continuous T-scores of depression, anxiety, hostility, and obsessions–compulsions. Partial correlations adjusted for sex were also examined. Multicollinearity was evaluated using variance inflation factors (VIF) prior to and within multivariable modelling.

The psychometric properties of the SCL-ADHD were further examined. Internal consistency was assessed using Cronbach’s α coefficient (61). Evidence related to internal structure was examined through confirmatory factor analysis (CFA) using maximum-likelihood estimation. Model fit was evaluated based on multiple indices (χ^2^/df ratio, Comparative Fit Index (CFI), Tucker–Lewis Index (TLI), Root Mean Square Error of Approximation (RMSEA), and Standardized Root Mean Square Residual (SRMR). Interpretation of model fit followed the criteria proposed by Schermelleh-Engel et al. (48), whereby RMSEA and SRMR values below 0.05 and CFI and TLI values above 0.95 indicate good fit, and RMSEA and SRMR values between 0.05 and 0.08 and CFI and TLI values between 0.90 and 0.95 reflect acceptable fit. In addition to structural validity, complementary construct-related evidence was explored was explored in line with COSMIN recommendations (62). Construct-related associations (rather than independent convergent validation) were examined by assessing associations between SCL-ADHD scores and conceptually related symptom dimensions derived from the SCL-90-R, including depression, anxiety, hostility, and obsessions–compulsions. Because these dimensions originate from the same assessment framework, these analyses were interpreted as evidence of internal coherence and expected symptom co-occurrence, and may be influenced by shared method variance. Discriminant validity was indirectly evaluated by examining weaker or non-significant associations with less theoretically related variables. Known-groups validity was explored by comparing SCL-ADHD classifications across subgroups expected to differ in ADHD symptomatology, such as sex and clinical risk profiles, while recognizing that these comparisons pertain to screening-positive status rather than clinically confirmed ADHD and should be considered exploratory and hypothesis-generating. These analyses were considered exploratory and hypothesis-generating, given the absence of an external gold-standard ADHD diagnosis in the present study. Because this study represents a secondary analysis of data originally collected for a broader mental health survey, no gold-standard diagnostic assessment for ADHD (e.g., structured clinical interview based on DSM or ICD criteria) was administered at the time of data collection. Consequently, criterion-related validity could not be examined, and all validity analyses were limited to internal structure and construct-related evidence.

All analyses were two-tailed, with statistical significance for inferential tests established at p < 0.05. Variable retention in multivariable models was based on independent contribution to overall model fit rather than on bivariate significance alone. The complete analytic workflow was systematically documented to ensure transparency and reproducibility. Statistical analysis syntax files can be made available from the corresponding author upon reasonable request, in accordance with ethical restrictions governing correctional data.

## Results

### Sociodemographic characteristics

A total of 836 incarcerated individuals participated in the study, of whom 621 were men (74.3%) and 215 were women (25.7%). The mean age of the total sample was 32.36 years (SD = 10.04), with no statistically significant sex differences. Most participants were Paraguayan nationals (97.7%), and 2.3% were foreign nationals. Regarding education, 14.2% had completed primary education, 20.0% secondary, and 1,7% had no formal education. A large proportion (69.4%) reported having children, and 92.0% indicated having had some form of paid employment before imprisonment. Monthly income prior to incarceration was below the national minimum wage for 46.4% of participants. Sociodemographic data are presented in **Table 1**.

**Table 1:** Sociodemographic characteristics of participants (N = 836).

Characteristics	Women (n = 215)		Men (n = 621)		Total	
	n	%	n	%	n	%
Age						
18 – 38	143	67.9	494	79.6	637	76.2
39 – 58	60	27.9	115	18.5	175	20.9
≥ 59	9	4.2	12	1.9	21	2.5
Nationality						
Paraguayan	210	97.7	607	97.7	817	97.7
Foreign national	5	2.3	14	2.3	19	2.3
Children						
None	30	14.0	226	36.4	256	30.6
1 – 5	154	71.5	384	61.8	538	64.4
6 – 10	30	14.0	8	1.3	38	4.5
11+	1	0.5	3	0.5	4	0.5
Education level						
Primary complete	40	18.6	79	12.7	119	14.2
Primary incomplete	34	15.8	130	20.9	164	19.6
Secondary complete	42	19.5	125	20.1	167	20.0
Secondary incomplete	72	33.5	241	38.8	313	37.4
No formal education	1	0.5	13	2.1	14	1.7
University complete	7	3.3	12	1.9	19	2.3
University incomplete	19	8.8	21	3.4	40	4.8
Worked before imprisonment						
Yes	180	83.7	589	94.8	769	92.0
No	35	16.3	32	5.2	67	8.0
Monthly income (n = 769)^*^						
Below minimum wage	88	48.9	269	45.7	357	46.4
Minimum wage	55	30.6	170	28.9	225	29.3
Above minimum wage	37	20.6	150	25.5	187	24.3

Percentages were calculated using the column-specific denominators (Women n = 215; Men n = 621; Total N = 836). ^*^Monthly income categories refer to self-reported income relative to the Paraguayan national minimum wage at the time of assessment, prior to incarceration. Income data (n = 769 of 836 participants) include only individuals reporting any monetary income; percentages are calculated within this subgroup.

### Prison context

Among the participants, 27.3% were serving sentences of more than five years, 24.9% had sentences of five years or less, and 47.8% were in pre-trial detention. Regarding the primary reason for imprisonment, 76.7% were convicted for violent crimes and 20.7% for drug-related offences. About 1% were in a maximum-security regime, and 30.0% reported having a family member also imprisoned. Prior incarcerations were reported by 53.2% of participants. The mean age at first incarceration was 25.53 years (SD = 10.1), and the median duration of current imprisonment was 3.8 years (IQR = 2.1–6.5). These data are detailed in **Table 2**.

**Table 2:** Prison context characteristics of participants (N = 836).

Characteristics	Women (n = 215)		Men (n = 621)		Total	
	n	%	n	%	n	%
Legal status						
Sentenced > 5 years	31	14.4	197	31.7	228	27.3
Sentenced ≤ 5 years	40	18.6	168	27.1	208	24.9
Remanded	144	67	256	41.2	400	47.8
Reason for imprisonment						
Violent offences	137	63.7	504	81.2	641	76.7
Drug-related offences	71	33	102	16.4	173	20.7
Sexual offences	5	2.3	34	5.5	39	4.7
Other	2	1	19	3.1	21	2.5
Maximum-security regime						
Yes	8	3.7	0	0	8	1.0
No	207	96.3	621	100	828	99.0
Family member in prison						
None	145	67.4	440	70.9	585	70.0
Sibling	30	14	83	13.4	113	13.5
Child	17	7.9	3	0.5	20	2.4
Other	6	2.8	19	3.1	25	3.0
Parent	3	1.4	11	1.8	14	1.7
Partner	9	4.2	2	0.3	11	1.3
Cousin	2	0.9	58	9.3	60	7.2
Niece/nephew	3	1.4	5	0.8	8	1.0
Prior incarcerations (recidivism)						
Yes	69	32.1	376	60.5	445	53.2
No	146	67.9	245	39.5	391	46.8
Time in prison (years)						
<2 years	180	83.7	387	62.3	567	67.8
2–7 years	31	14.4	181	29.1	212	25.4
7–12 years	1	0.5	35	5.6	36	4.3
12–20 years	3	1.4	18	2.9	21	2.5
Age at first incarceration						
12 – 38	165	76.7	579	93.3	744	89.0
39 – 58	43	20	38	6.1	81	9.7
≥ 59	7	3.3	4	0.6	11	1.3

Percentages were calculated using column-specific denominators (Women n = 215; Men n = 621; Total N = 836). Legal status categories refer to sentence length at the time of assessment. Remanded indicates pre-trial detention. Maximum-security regime refers to administrative classification within the correctional system. Prior incarcerations indicate at least one previous imprisonment before the current sentence.

### Health-related data

Regarding physical health, 25.4% of participants reported at least one medical condition, the most common being hypertension and gastrointestinal problems. Psychiatric history was reported by 4.2% of the sample, and 17.0% were currently receiving some form of treatment, most frequently psychological or psychiatric care. No significant differences were observed between men and women in these variables. Full health-related data are summarized in **Table 3**.

**Table 3:** Health-related characteristics of participants (N = 836).

Characteristics	Women (n = 215)		Men (n = 621)		Total	
	n	%	n	%	n	%
Self-reported medical condition/history						
Yes	76	35.3	136	21.9	212	25.4
No	139	64.7	485	78.1	624	74.6
Self-reported mental health diagnosis/history						
Yes	22	10.2	13	2.4	35	4.2
No	193	89.8	608	97.6	801	95.8
Currently receiving medical treatment						
Yes	52	24.2	90	14.5	142	17.0
No	163	75.8	531	85.5	694	83.0
Currently receiving psychological/psychiatric treatment						
Yes	35	16.3	21	3.4	56	6.7
No	180	83.7	600	96.6	780	93.3

Percentages are column percentages calculated using sex-specific denominators (Women n = 215; Men n = 621; Total N = 836). “Self-reported medical condition/history” and “self-reported mental health diagnosis/history” refer to conditions reported by participants at the time of assessment. Treatment variables indicate current treatment during incarceration.

### Mental health characteristics

According to the C-SSRS, 24.8% of participants reported suicidal ideation at some point during imprisonment, and 3.8% reported at least one suicide attempt. Alcohol consumption was more frequent among women (36.7%), as was drug use (45.6%). Problematic alcohol use (MULTICAGE CAD-4) was identified in 50.5% of cases, and problematic substance use in 61.9%. The SCL-90-R showed excellent internal consistency in this sample (Cronbach’s α = 0.96). Regarding the symptomatic dimensions of the SCL-90-R, the highest percentages of screening-based at-risk classifications were observed in depression, anxiety, and psychoticism, with no marked differences between sexes. Detailed distributions of mental health variables are shown in **Table 4**.

**Table 4:** Mental health characteristics of participants (N = 836).

Characteristics	Women (n = 215)		Men (n = 621)		Total	
	n	%	n	%	n	%
Suicide risk^¥^						
High	56	26.0	30	4.8	86	10.3
Moderate	3	1.4	33	5.3	36	4.3
Low	27	12.6	58	9.3	85	10.2
None	129	60.0	500	80.5	629	75.2
Alcohol consumption (yes)	79	36.7	129	20.8	208	24.9
Alcohol-related problems^*^ (n = 208)	47	59.5	58	44.9	105	50.5
Drug use (yes)	98	45.6	235	37.8	333	39.8
Drug-related problems^**^ (n = 333)	72	73.5	134	57.0	206	61.9
Symptom dimensions^¥¥^						
Somatization	46	21.4	146	23.5	192	23.0
Obsessions and compulsions	7	3.3	89	14.3	96	11.5
Interpersonal sensitivity	27	12.6	113	18.2	140	16.7
Depression	51	23.7	125	20.1	176	21.1
Anxiety	29	13.5	127	20.5	156	18.7
Hostility	39	18.1	87	14.0	126	15.1
Phobic anxiety	17	7.9	158	25.4	175	20.9
Paranoid ideation	41	19.1	135	21.7	176	21.1
Psychoticism	34	15.8	175	28.2	209	25.0

Percentages are column percentages calculated using sex-specific denominators (Women n = 215; Men n = 621; Total N = 836). ^¥^Suicide risk assessed according to the Columbia–Suicide Severity Rating Scale (C-SSRS). ^*^Alcohol-related problems were defined as a score ≥ 2 on the alcohol subscale of the MULTICAGE CAD-4 and percentages were calculated among participants reporting alcohol consumption within each sex. ^**^Drug-related problems were defined as a score ≥ 2 on the drug subscale of the MULTICAGE CAD-4 and percentages were calculated among participants reporting drug use within each sex. ^¥¥^Symptom dimensions classified as “at-risk” according to the SCL-90-R manual (T-score ≥ 63 on the respective dimension); these classifications indicate elevated symptom burden and do not constitute formal psychiatric diagnoses.

### Psychometric properties of the SCL-ADHD

The nine-item SCL-ADHD subscale exhibited strong psychometric properties in the incarcerated population. Internal consistency was good (Cronbach’s α = 0.758), and both Bartlett’s test of sphericity (χ^2^ = 1299.9, p < 0.001) and the Kaiser–Meyer–Olkin index (KMO = 0.823) confirmed the adequacy of the correlation matrix for factor analysis.

The model was significant (χ^2^ = 115.77, p < 0.001), with fit indices of CFI = 0.928, TLI = 0.892, GFI = 0.979, NFI = 0.911, RMSEA = 0.068, and SRMR = 0.037. According to the criteria of Schermelleh-Engel et al. (48), RMSEA and SRMR indicate acceptable fit, and the CFI falls within the acceptable range. Although the TLI was marginally below the 0.90 threshold, the overall pattern of indices supports an acceptable three-factor solution. All items loaded significantly on their corresponding factors (p < 0.001), as shown in **Table 5**.

**Table 5:** Standardised factor loadings from confirmatory factor analysis (maximum-likelihood estimation) for the three-factor SCL-ADHD model (N = 836).

Factor/Item	Standardized loading (λ)	p-value
Factor 1 (Nervousness)		
Item 2	0.818	< 0.001
Item 57	0.868	< 0.001
Item 78	0.719	< 0.001
Factor 2 (Cognitive impairment)		
Item 9	0.734	< 0.001
Item 28	0.557	< 0.001
Item 55	0.661	< 0.001
Factor 3 (Irritability)		
Item 11	0.873	< 0.001
Item 24	0.759	< 0.001
Item 74	0.709	< 0.001

Standardised factor loadings (λ) derived from maximum-likelihood estimation. All loadings were statistically significant at p <.001. Items correspond to the original SCL-90-R item numbering used to derive the SCL-ADHD subscale.

### Prevalence and associated factors of screening-positive ADHD status

Overall, 33.4% of participants scored above the threshold for screening-positive ADHD status. The difference between sexes was statistically significant (χ^2^ = 4.22; p = 0.04; OR = 1.40, 95% CI = 1.02–1.93), indicating a higher prevalence of screening-positive ADHD status among women. Specifically, 84 of the 215 women (39.1%) met criteria for screening-positive ADHD status, compared with 195 of the 621 men (31.4%).

No significant independent associations were observed between screening-positive ADHD status and legal status, type of offence, or recidivism after multivariable adjustment. Legal status showed a modest association with screening-positive ADHD status in bivariate analyses (**Table 6**), but this association did not remain significant in the adjusted model. Suicidal risk, however, was markedly higher in the screening-positive group (OR = 3.85; p < 0.001). Regarding psychopathological dimensions, all SCL-90-R domains showed significant associations with screening-positive ADHD status. The strongest odds ratios were observed for hostility (OR = 43.5), anxiety (OR = 21.3), obsessions–compulsions (OR = 27.8), and depression (OR = 11.8), all p < 0.001. Additional significant differences were found for medical conditions (OR = 1.7; p = 0.001) and previous employment before imprisonment (OR = 0.55; p = 0.02), whereas family history and previous mental illness were not statistically significant (**Table 6**).

**Table 6:** Bivariate associations between participant characteristics and screening-positive ADHD status (N = 836).

Characteristics	Screening-positive ADHD status (n=279)		Screening-negative ADHD status (n=557)		Total		OR	p-value
	n	%	n	%	n	%		
Sex								
Women	84	30.1	131	23.5	215	25.7	1.4	0.04
Men	195	69.9	426	76.5	621	74.3		
Legal status								
Sentenced > 5 years	73	26.2	155	27.8	228	27.3	NA	0.028
Sentenced ≤ 5 years	56	20.1	152	27.3	208	24.9		
Remanded	150	53.8	250	44.9	400	47.8		
Reason for imprisonment								
Violent offences	220	78.9	421	75.6	641	76.7	1.20	0.33
Drug-related offences	55	19.7	118	21.2	173	20.7	1.09	0.62
Sexual offences	14	5.0	25	4.5	39	4.7	0.89	0.732
Prior incarcerations (recidivism)								
Yes	145	52.0	300	53.9	443	53.0	0.93	0.542
No	134	48.0	257	46.1	391	46.8		
Suicide risk^¥^								
Yes	118	42.3	89	16.0	207	24.8	3.85	< 0.001
No	161	57.7	468	84.0	629	75.2		
Symptom dimensions^¥¥^								
Somatization	129	46.2	63	11.3	192	23.0	6.74	< 0.001
Obsessions and compulsions	87	31.2	9	1.6	96	11.5	27.8	< 0.001
Interpersonal sensitivity	107	38.4	33	5.9	140	16.7	9.9	< 0.001
Depression	135	48.4	41	7.4	176	21.1	11.76	< 0.001
Anxiety	133	47.7	23	4.1	156	18.7	21.28	< 0.001
Hostility	117	41.9	9	1.6	126	15.1	43.5	< 0.001
Phobic anxiety	116	41.6	59	10.6	175	20.9	6.02	< 0.001
Paranoid ideation	123	44.1	53	9.5	176	21.1	7.52	< 0.001
Psychoticism	139	49.8	70	12.6	209	25.0	6.9	< 0.001
Worked before imprisonment								
Yes	248	88.9	521	93.5	769	92.0	0.55	0.02
No	31	11.1	36	6.5	67	8.0		
Family member in prison								
Yes	83	29.7	168	30.2	251	30.0	0.98	0.902
No	196	70.3	389	69.8	585	70.0		
Self-reported medical condition								
Yes	90	32.3	122	21.9	212	25.4	1.7	0.001
No	189	67.7	435	78.1	624	74.6		
Self-reported mental health diagnosis								
Yes	17	6.1	18	3.2	35	4.2	1.94	0.051
No	262	93.9	539	96.8	801	95.8		
Alcohol-related problems^*^								
Yes	54	19.4	51	9.2	105	12.6	2.46	< 0.001
No	179	64.2	416	74.7	595	71.2		
Drug-related problems^**^								
Yes	92	33.0	114	20.5	206	24.6	1.97	< 0.001
No	149	53.4	364	65.4	513	61.4		

Percentages are column percentages within screening-positive and screening-negative groups. Odds ratios (OR) are unadjusted estimates derived from bivariate logistic regression analyses with the absence of the characteristic serving as the reference category unless otherwise specified. NA = not calculable due to sparse cells in the corresponding comparison. Screening-positive ADHD status was defined as SCL-ADHD ≥ 12 and does not constitute a clinical diagnosis of Attention-Deficit/Hyperactivity Disorder. ^¥^Suicide risk assessed according to the Columbia–Suicide Severity Rating Scale (C-SSRS). ^¥¥^Symptom dimensions classified as “at-risk” according to the SCL-90-R manual (T-score ≥ 63 on the respective dimension); these classifications indicate elevated symptom burden and do not constitute formal psychiatric diagnoses. ^*^Alcohol-related problems were defined as a score ≥ 2 on the alcohol subscale of the MULTICAGE CAD-4 and analyses were restricted to participants reporting alcohol consumption. ^**^Drug-related problems were defined as a score ≥ 2 on the drug subscale of the MULTICAGE CAD-4 and analyses were restricted to participants reporting drug use. p < 0.05 indicates statistical significance.

In the binary logistic regression model, variables that independently contributed to overall model fit, as evaluated by likelihood-ratio tests and AIC comparisons, were retained. The final model demonstrated excellent overall fit (χ^2^(5) = 430; *p* < 0.001; AIC = 647; McFadden’s R^2^ = 0.403), with no collinearity problems (VIF < 1.1). Being female was independently associated with higher odds of screening-positive ADHD status (aOR = 2.06; 95% CI = 1.33–3.19; *p* = 0.001). Likewise, scoring within the “at-risk” range on the SCL-90-R dimensions of depression (aOR = 2.77; 95% CI = 1.62–4.74), anxiety (aOR = 8.62; 95% CI = 4.79–15.48), hostility (aOR = 24.89; 95% CI = 11.65–53.15), and obsessions–compulsions (aOR = 8.71; 95% CI = 3.68–20.63) was independently associated with greater likelihood of screening-positive ADHD status (**Table 7**). The model explained approximately 40% of the variance in screening-positive ADHD status. Although several variables showed significant associations with screening-positive ADHD status in bivariate analyses (**Table 6**), only those contributing independently to the multivariable model were retained after evaluation of overall model fit. Correctional context variables (legal status, prior incarcerations, age at first imprisonment, duration of current incarceration, and maximum-security regime) were evaluated as candidate predictors but did not independently contribute to the final multivariable model.

**Table 7:** Binary multivariable logistic regression model for factors associated with screening-positive ADHD status according to the SCL-ADHD (N = 836).

Predictor (reference)	B (SE)	aOR	95% CI	p-value
Sex (female)	0.723 (0.224)	2.06	1.33 – 3.19	0.001
Depression (at-risk)	1.018 (0.274)	2.77	1.62 – 4.74	< 0.001
Anxiety (at-risk)	2.154 (0.299)	8.62	4.79 – 15.48	< 0.001
Hostility (at-risk)	3.214 (0.387)	24.89	11.65 – 53.15	< 0.001
Obsessions–compulsions (at-risk)	2.164 (0.440)	8.71	3.68 – 20.63	< 0.001

aOR, adjusted odds ratio; SE, standard error; CI, confidence interval. Predictors were entered simultaneously in a multivariable logistic regression model following a two-stage exploratory selection strategy as described in the Methods section. Symptom dimensions were entered as dichotomous variables (“at-risk” vs. non–at-risk; T-score ≥ 63), with the non–at-risk category serving as the reference group. For sex, male participants served as the reference category. Model fit was evaluated using the omnibus likelihood-ratio test and the Akaike Information Criterion (AIC). p < 0.05 was considered statistically significant.

To evaluate the robustness of the associations and address potential inflation of effect sizes due to dichotomization, a sensitivity analysis was conducted in which SCL-90-R symptom dimensions were entered as continuous standardized T-scores rather than dichotomized “at-risk” variables. In this alternative specification, screening-positive ADHD status remained independently associated with female sex (aOR = 3.77; 95% CI: 2.12–6.68; p < 0.001), as well as with higher T-scores for anxiety (aOR = 1.14; 95% CI: 1.11–1.18; p < 0.001), hostility (aOR = 1.15; 95% CI: 1.12–1.19; p < 0.001), and obsessions–compulsions (aOR = 1.11; 95% CI: 1.07–1.15; p < 0.001). In contrast, depression T-scores did not retain independent significance after adjustment (p = 0.135). Importantly, no evidence of multicollinearity was observed (VIF range: 1.02–1.53), and the number of outcome events per predictor (EPV = 55.8) indicated a low risk of model overfitting. These findings suggest that the observed associations are not solely attributable to dichotomization or model instability (**Table 8**).

**Table 8:** Sensitivity analysis: Binary multivariable logistic regression model using continuous SCL-90-R T-scores for factors associated with screening-positive ADHD status (N = 836).

Predictor (reference)	B (SE)	aOR	95% CI	p-value
Sex (female)	1.3260 (0.2925)	3.766	2.123–6.680	p<0.001
Depression (T-score)	−0.0236 (0.0158)	0.977	0.947–1.010	0.135
Anxiety (T-score)	0.1337 (0.0171)	1.143	1.105–1.180	p<0.001
Hostility (T-score)	0.1426 (0.0157)	1.153	1.118–1.190	p<0.001
Obsessions–compulsions (T-score)	0.1049 (0.0185)	1.111	1.071–1.150	p<0.001

aOR, adjusted odds ratio; SE, standard error; CI, confidence interval. Outcome: screening-positive ADHD status (SCL-ADHD ≥ 12 vs < 12). This sensitivity model replicated the multivariable specification of **Table 7** but entered SCL-90-R symptom dimensions as continuous standardized T-scores rather than dichotomized “at-risk” variables. Therefore, aORs represent the change in odds of screening-positive ADHD status per 1-point increase in standardized symptom severity. For sex, male participants served as the reference category. Collinearity diagnostics indicated no relevant multicollinearity (VIF range: 1.02–1.53). The final model included five predictors with 279 screening-positive cases (EPV = 55.8), indicating low risk of overfitting.

To further examine potential construct overlap and shared method variance, Pearson correlations among continuous SCL-90-R T-scores (depression, anxiety, hostility, and obsessions–compulsions) were estimated in the total sample (N = 836). All correlations were positive and statistically significant (p <.001), ranging from moderate to high magnitude (r = 0.574–0.776). The strongest association was observed between depression and anxiety (r = 0.776; 95% CI: 0.748–0.802), whereas the remaining coefficients were in the moderate range. Although correlations were substantial, they remained below levels typically associated with severe multicollinearity. When entered simultaneously into regression models, variance inflation factors ranged from 1.84 to 3.16, within conventional acceptability thresholds (VIF < 5), indicating no evidence of problematic multicollinearity. Exploratory partial correlations adjusted for sex yielded very similar coefficients, suggesting that the observed associations were not substantially driven by sex differences. Together, these findings support model stability; however, the possibility of shared method variance inherent to symptom measures derived from the same assessment framework cannot be entirely excluded. Full correlation matrices and VIF estimates are provided in the **Supplementary Material** to enhance transparency and reproducibility.

## Discussion

### Overview of findings

This study provides the first national-level empirical evidence on ADHD symptoms among incarcerated individuals in Paraguay, integrating epidemiological, clinical, and psychometric perspectives. Approximately one-third of participants met the threshold for screening-positive ADHD (33.4%), a prevalence comparable to global estimates of 20–30% among prison populations reported in meta-analyses (13, 14, 18). Importantly, ADHD status in this study was defined through a validated screening instrument rather than a formal clinical diagnosis. The SCL-ADHD is designed to detect elevated ADHD symptom burden, but it does not replace a comprehensive diagnostic assessment based on developmental history and structured clinical interview. Accordingly, all findings should be interpreted as associations with screened ADHD symptomatology rather than with confirmed ADHD diagnoses. A formal diagnosis of adult ADHD requires evidence of symptom onset before age 12, cross-situational persistence, clinically significant impairment, and exclusion of alternative explanations, criteria that cannot be fully reconstructed using cross-sectional screening data (39).

Recent forensic longitudinal evidence suggests that ADHD in justice-involved populations is underdiagnosed and frequently diagnostically displaced over time. A retrospective cohort study from Switzerland following offenders with a confirmed childhood ADHD diagnosis demonstrated substantial diagnostic instability in adulthood, with ADHD often being discontinued or replaced by personality disorder diagnoses in forensic expert assessments and treatment reports (63). Notably, these diagnostic shifts did not consistently correspond to systematic ADHD reassessments or documented symptom remission, but appeared to reflect contextual, interpretative, or institutional factors within forensic settings. These findings support the interpretation that elevated ADHD symptom burden observed in prison populations cannot be adequately explained by incarceration-related stress alone. Instead, they point toward a structural vulnerability of ADHD to being “lost” within adult forensic psychiatry, particularly when symptom presentations overlap with antisocial traits or emotional dysregulation. In this light, screening-based identification of screening-positive ADHD in prison settings may serve as a corrective signal against diagnostic overshadowing.

The differential interpretation of screening-positive ADHD symptoms in correctional settings warrants particular caution. High rates of trauma exposure, substance dependence, mood instability, and personality pathology in incarcerated populations create substantial symptom overlap (13, 64). Hyperarousal and concentration difficulties may reflect PTSD-related processes, while impulsivity and irritability may be embedded within affective or personality-related dysregulation. Given the cross-sectional design and reliance on symptom-based screening, the present findings cannot disentangle neurodevelopmental ADHD from incarceration-related distress or comorbid psychiatric conditions. Therefore, the observed prevalence should be understood as reflecting elevated ADHD-like symptomatology within a diagnostically complex environment rather than confirmed adult ADHD.

ADHD symptoms were more frequent among women than among men in the sample: 39.1% of women and 31.4% of men screened positive, and female sex remained an independent correlate of screening-positive ADHD status after adjustment for psychopathological dimensions. Although this finding may appear unexpected given the historically higher prevalence of ADHD reported among males in community samples, it is increasingly consistent with emerging evidence from correctional settings. Several factors may help contextualize this pattern.

First, ADHD in females is more likely to manifest through internalising symptoms, emotional dysregulation, and affective instability rather than overt hyperactivity, which may lead to under-recognition in community and clinical contexts but greater symptom salience in high-stress environments such as prisons. Prior research suggests that incarcerated women with ADHD often present with higher rates of comorbid mood disorders, trauma exposure, self-harm, and suicidality, which may amplify symptom reporting during incarceration (28, 29). Second, the prison environment itself may differentially impact women, intensifying emotional distress and emotional dysregulation in a population already exposed to cumulative psychosocial adversity. Evidence indicates that incarcerated women experience higher levels of trauma history, interpersonal violence, caregiving disruption, and separation-related stress than their male counterparts, factors that are known to exacerbate ADHD-related difficulties in emotion regulation, impulse control, and stress tolerance (64–66). Within such contexts, ADHD symptoms may become more clinically salient among women than men, even in the absence of differences in underlying neurodevelopmental vulnerability.

Finally, detection bias should be considered. Screening instruments such as the SCL-ADHD may be particularly sensitive to the affective and cognitive dimensions of ADHD, such as emotional lability, concentration difficulties, and subjective distress, which are more characteristic of female ADHD presentations (67, 68). This measurement sensitivity, combined with higher levels of comorbid internalising psychopathology, may contribute to higher observed prevalence estimates among incarcerated women when symptom-based screening tools are used. Taken together, these findings emphasize the importance of gender-sensitive approaches to ADHD screening and intervention in correctional mental health services.

The associations observed between screening-positive ADHD status and psychopathological dimensions, particularly hostility, anxiety, and obsessive–compulsive symptoms, should be interpreted cautiously and are consistent with evidence suggesting that ADHD in correctional contexts frequently co-occurs with emotional dysregulation and elevated suicide risk (69, 70). Rather than indicating independent or causal effects, these associations likely reflect overlapping symptom burden within a dimensional psychopathology framework and partially shared variance across symptom domains. These findings align with the broader literature showing that untreated ADHD is associated with impulsivity, stress reactivity, and comorbid affective disturbances, which have been linked to institutional difficulties and adverse outcomes.

The magnitude of the association observed for hostility should be interpreted cautiously, as it likely reflects the strong clustering of screening-positive ADHD symptoms with emotional dysregulation and externalizing psychopathology rather than a direct causal effect. Emotional dysregulation (encompassing affective instability, irritability, and hostility) has been documented as a prominent dimension in adults with ADHD, with implications for severity, comorbidity, and functional impairment (71–73). Both ADHD screening classification and psychopathological dimensions were derived from the same self-report instrument (SCL-90-R), raising the possibility of shared method variance and construct overlap. This dimensional perspective on ADHD aligns with evidence that aggressive and hostility-related traits frequently co-occur with core ADHD symptoms, particularly in high-stress contexts such as incarceration. Importantly, sensitivity analyses using continuous symptom scores, as well as correlation and collinearity diagnostics, indicated that the observed associations were not solely attributable to dichotomization or statistical instability. Nevertheless, given the common measurement framework, some degree of shared variance cannot be fully excluded and should be considered when interpreting effect magnitudes.

Taken together, the regression findings should be interpreted strictly as cross-sectional associations rather than causal relationships. The identified correlates do not imply temporal directionality, and it cannot be determined whether elevated ADHD symptom burden contributes to higher levels of anxiety, hostility, or obsessive–compulsive symptoms, or whether these dimensions reflect overlapping or mutually reinforcing psychopathological processes within a shared vulnerability framework. Given the secondary, cross-sectional design and reliance on screening-based measures, the multivariable model identifies independent associations rather than etiological determinants.

In addition to the specific symptom dimensions examined in this study, the observed pattern is consistent with a broader body of literature documenting high rates of psychiatric comorbidity among adults with ADHD. Meta-analytic and large-scale clinical studies have consistently shown that ADHD in adulthood is frequently accompanied by anxiety disorders, mood disorders, substance use disorders, and obsessive–compulsive symptoms, reflecting a shared vulnerability to emotional dysregulation and impaired executive control rather than discrete diagnostic entities (37, 38, 74). In correctional populations, this comorbidity profile appears particularly pronounced, with screening-positive ADHD status often co-occurring with affective instability, trauma-related symptoms, suicidality, and externalizing psychopathology, which together contribute to functional impairment and clinical complexity. These findings support dimensional models of adult psychopathology, in which ADHD represents a core neurodevelopmental condition embedded within broader internalizing and externalizing spectra, rather than an isolated disorder (13, 64, 69, 75).

In our study, no significant associations were observed between screening-positive ADHD status and criminological variables such as offence type, legal status, or recidivism. Although this finding may appear to diverge from parts of the existing literature, it is largely consistent with evidence suggesting that cross-sectional assessments of current ADHD symptom burden are more strongly related to contemporaneous psychological distress than to distal criminological outcomes. Previous longitudinal and register-based studies linking ADHD to criminal trajectories have primarily relied on developmental histories, age at first offence, or long-term follow-up data, which were not available in the present secondary analysis (18, 19, 26). Importantly, the present study assessed current symptomatology rather than life-course patterns of offending, which may partly account for the absence of independent criminological associations. Moreover, the prison environment itself may attenuate behavioural differentiation related to offence characteristics while amplifying internalising symptoms and emotional dysregulation, potentially explaining the predominance of psychological rather than criminological correlates in this sample.

### Psychometric and methodological contributions

Beyond prevalence estimates, this investigation extends the application of the SCL-ADHD to a Spanish-speaking correctional population, providing initial psychometric evidence for its use in this context as a screening measure of ADHD symptom burden. Prior prison studies have relied primarily on structured diagnostic interviews such as the MINI or DIVA-5, which, although diagnostically robust, are resource-intensive and difficult to implement systematically in overcrowded or under-resourced correctional settings (14, 64). The SCL-ADHD, originally developed by Eich et al. (9), offers a brief screening approach that may be pragmatically integrated into routine mental health assessments.

In the present study, the SCL-ADHD demonstrated good internal consistency and a factorial structure consistent with the model identified in the present analysis. The three-factor configuration observed in the present sample is conceptually consistent with the structure reported by Eich et al. (9), who described correlated dimensions labeled Nervousness, Cognitive Impairment, and Irritability. Although factor labels may not be strictly interchangeable across samples, the present findings support a multidimensional representation of ADHD-related symptom burden as operationalized within this screening framework. Fit indices largely supported an acceptable three-factor solution; although the TLI was marginally below the conventional 0.90 threshold, the overall pattern of indices (CFI, RMSEA, and SRMR) indicated acceptable model fit according to established CFA criteria. All items were derived from the previously validated Spanish version of the SCL-90-R, and a structured semantic equivalence review was undertaken to ensure contextual appropriateness for Paraguayan Spanish. These findings support the internal coherence of the SCL-ADHD as a screening measure of current ADHD symptom burden and screening-positive ADHD classification in incarcerated adults. However, they should be interpreted as preliminary, as the present psychometric evaluation did not include gold-standard diagnostic interviews, test–retest reliability, or criterion validity analyses. Moreover, this evidence pertains to internal structure and expected associations within the same assessment framework, and may be influenced by shared method variance; therefore, it should not be interpreted as full construct validation in the absence of external diagnostic criteria.

Accordingly, the SCL-ADHD should be regarded as a screening tool rather than a diagnostic instrument. Its utility lies in identifying individuals with elevated ADHD symptomatology who screen positive for ADHD and may therefore warrant comprehensive clinical evaluation, particularly in settings where comprehensive diagnostic assessments are not feasible. Further studies incorporating structured diagnostic interviews across diverse Latin American correctional contexts are warranted to strengthen the evidence base for its broader application, as the present findings are limited to a Paraguayan prison sample.

In addition to its cross-sectional associations with impulsivity and emotional dysregulation, ADHD has been linked to adverse long-term criminal trajectories and recidivism. Large-scale cohort and follow-up studies indicate that individuals with ADHD accumulate more arrests, convictions, and incarcerations than those without ADHD (18, 19, 26). In contrast, pharmacoepidemiologic evidence suggests that evidence-based treatment can mitigate these risks. Register-based studies from Sweden and Denmark have shown that periods of ADHD medication are associated with substantial reductions in criminal convictions (i.e., approximately 32% among men and 41% among women) and with lower rates of violent reoffending after prison release (11, 12). Randomized and observational studies in offender populations further suggest that stimulant treatment and multimodal interventions can improve behavioural control and may reduce disciplinary incidents and recidivism (68, 76, 77). Against this backdrop, our finding that one-third of inmates (particularly women) screened positive for ADHD based on a standardized symptom screening instrument underlines a missed opportunity for effective, potentially criminologically relevant treatment in the Paraguayan prison system, provided that screening-positive cases undergo appropriate diagnostic confirmation and clinical assessment.

From a public health and criminal justice perspective, integrating ADHD assessment and treatment into routine prison mental health care is clinically justified and aligns closely with the Risk–Need–Responsivity (RNR) framework. Within this model, ADHD-specific interventions may enhance responsivity by improving attention, emotional regulation, and executive functioning, thereby facilitating engagement in correctional and rehabilitative programs (78), particularly when systematic screening is followed by comprehensive diagnostic evaluation and individualized treatment planning.

However, this theoretical alignment does not guarantee effective implementation in practice; the application of RNR-based rehabilitation in incarcerated individuals with ADHD or elevated ADHD symptom burden may be systematically challenged by institutional dynamics. Qualitative research on correctional staff perceptions suggests that inmates with ADHD or ADHD-like symptom presentations are frequently viewed as disruptive, non-compliant, and resource-intensive, with core symptoms such as impulsivity, distractibility, and emotional reactivity often interpreted as wilful misconduct rather than manifestations of a neurodevelopmental condition (79). As a consequence, affected individuals may be disproportionately exposed to disciplinary responses, including segregation or solitary confinement, which are likely to further exacerbate emotion dysregulation and behavioural instability (Buadze et al., 2021). These dynamics directly undermine the responsivity principle central to RNR-based interventions, indicating that reduced treatment effectiveness in this population may reflect contextual and systemic misalignment rather than individual treatment resistance.

Addressing ADHD-related impairments or elevated ADHD symptom burden identified through screening may also target criminogenic needs linked to impulsivity, poor self-control, and decision-making deficits, which are central mechanisms underlying persistent offending. Population-based and correctional studies underline how timely ADHD diagnosis, combined with pharmacological treatment and structured psychosocial interventions, including stimulant medication and cognitive-behavioural therapies adapted for ADHD, can reduce criminal behaviour, violent reoffending, and other adverse outcomes (11, 12, 68, 77, 80), once a formal diagnostic evaluation has confirmed the presence of the disorder.

By improving responsivity to rehabilitation and addressing underlying neurodevelopmental vulnerabilities, ADHD-focused interventions may have the potential to contribute to meaningful reductions in recidivism risk, particularly in high-risk correctional populations. Although the present study does not establish causal effects on criminal outcomes, aligning Paraguayan prison policies with existing international evidence through systematic screening, access to guideline-based pharmacological and psychotherapeutic treatment following appropriate diagnostic assessment, and continuity of care after release, could therefore support both improved mental health outcomes and broader public safety (70, 81).

### Strengths and limitations

This study has several strengths. It is the first nationwide investigation in Paraguay to examine screening-positive ADHD status in prison settings using probabilistic sampling across both male and female populations and employing a validated psychometric tool. The large sample size, rigorous data quality procedures, and psychometric analyses strengthen confidence in the findings. Additionally, adapting and validating the SCL-ADHD in Spanish constitutes a practical and methodological advance for Latin American research.

However, certain limitations must be acknowledged. This study was based on a secondary analysis of data originally collected for a broader epidemiological investigation of mental health in prisons, and therefore no gold-standard diagnostic assessment for ADHD (e.g., structured clinical interview) was available. The cross-sectional design precludes causal inferences, and ADHD classification relied on a self-report screening instrument rather than diagnostic interviews. Because symptoms were assessed at a single time point during incarceration, it was not possible to determine whether reported ADHD-related symptoms predated imprisonment, emerged during incarceration, or were exacerbated by the prison environment, resulting in temporal ambiguity regarding symptom onset and persistence. Although multivariable modelling was used to identify independent associations, residual confounding cannot be excluded, particularly in the absence of longitudinal developmental data. Crucially, key diagnostic criteria required for adult ADHD—such as evidence of childhood onset, cross-situational persistence, and functional impairment across developmental stages—could not be reconstructed within the present design. Information on age at ADHD onset, developmental trajectory, and prior diagnostic history was not available, limiting inferences regarding symptom persistence versus incarceration-related exacerbation. Furthermore, the absence of detailed developmental and life-course criminological data (e.g., age at first offence, persistence of offending, childhood behavioural history) limits the ability to detect more distal criminological associations that have been described in longitudinal studies.

Moreover, several symptom domains assessed in this study (e.g., inattention, irritability, hyperarousal, emotional dysregulation) overlap with trauma-related symptoms, substance-related effects, mood disorders, and certain personality pathology frequently observed in correctional populations. In the absence of structured differential diagnostic assessment, some cases classified as screening-positive ADHD may reflect incarceration-related distress or comorbid psychopathology rather than a primary neurodevelopmental disorder. Additionally, reliance on self-report measures in a prison context introduces the possibility of response-style effects, including symptom over-reporting, exaggeration related to perceived secondary gain, or heightened distress expression in environments characterized by psychological strain.

Although internal consistency and factorial validity were robust, further studies are needed to assess sensitivity and specificity in Latin American correctional settings. In addition, while all items were derived from a previously validated Spanish version of the SCL-90-R, no formal forward–backward translation procedure was conducted for the present contextual adaptation. Although the semantic equivalence review supports conceptual alignment, the absence of formal back-translation should be considered when interpreting cross-linguistic comparability. The cut-off applied was derived from a clinical outpatient validation sample, and its operating characteristics in correctional populations with high psychiatric comorbidity remain to be fully established. Finally, although random sampling minimized bias, the results may not fully generalize to smaller regional prisons or populations with distinct sociodemographic characteristics. Findings should therefore be interpreted within the specific institutional and cultural context of the Paraguayan correctional system and may not be directly transferable to jurisdictions with different sentencing structures, prison conditions, or mental health service configurations.

Beyond these study-specific limitations, several methodological constraints inherent to prison-based research should be acknowledged. Psychopathological dimensions and ADHD symptoms were assessed using self-report instruments at a single time point and may have been influenced by current emotional distress, incarceration-related stressors, and shared method variance. Moreover, the examination of construct-related associations relied largely on symptom dimensions derived from the same assessment framework (SCL-90-R), which may inflate correlations due to shared measurement characteristics. Convergent findings should therefore be interpreted as evidence of internal coherence rather than independent cross-instrument validation. Although sensitivity analyses and collinearity diagnostics did not indicate problematic multicollinearity, the possibility of construct overlap within a common measurement framework cannot be entirely excluded.

Information on prior treatment exposure, medication status, and engagement in rehabilitative programs was not available, precluding analyses of treatment effects or responsivity outcomes. Finally, institutional, cultural, and systemic characteristics of the Paraguayan prison system may shape the expression and reporting of symptoms, underscoring the need for cautious interpretation and for replication in other correctional contexts.

### Implications for practice and future directions

Recent longitudinal work in a Swiss forensic outpatient cohort has highlighted that ADHD diagnoses are frequently discontinued along the criminal justice pathway (63). In that study, only a small minority of patients with documented childhood ADHD retained the diagnosis into adulthood, whereas many were later relabelled with personality or substance use disorders despite persisting neurodevelopmental symptoms (63). These “lost diagnoses” are clinically and criminologically relevant, as they may delay access to targeted ADHD treatment, shift the focus toward less effective interventions, and ultimately influence risk management and reoffending patterns.

In light of the current data—based on screening-positive ADHD status rather than confirmed diagnoses—systematic screening at prison entry and structured diagnostic follow-up appear crucial to prevent diagnostic erosion and to ensure that affected individuals can benefit from disorder-specific interventions when clinically indicated.

## Conclusions

This study provides the first empirical evidence on the prevalence, psychological correlates, and psychometric assessment of screening-positive ADHD symptoms among incarcerated individuals in Paraguay. The high proportion of individuals meeting the SCL-ADHD cut-off, together with strong associations with emotional dysregulation, hostility, and suicidality, highlights the importance of early detection and careful clinical evaluation in correctional settings. Given the screening-based nature of the classification and the cross-sectional design, findings should not be interpreted as indicating confirmed ADHD diagnoses, but rather as identifying a subgroup with elevated ADHD symptom burden requiring further assessment.

The present psychometric findings regarding the SCL-ADHD in Spanish represent a preliminary methodological contribution that may inform future research and practice in correctional mental health in Latin America, while remaining specific to the Paraguayan context. Overall, ADHD-related symptom burden in prisons should be recognized as a relevant public health and justice issue, requiring coordinated responses that integrate screening, differential diagnosis, and access to evidence-based interventions.

## Data Availability

The raw data supporting the conclusions of this article will be made available by the authors, without undue reservation.

## Appendix I Semantic equivalence of SCL-ADHD subscale items across original English, German, and validated Spanish SCL-90-R versions (9).

**Table:** This table documents the semantic equivalence of the nine SCL-ADHD items across the original English and German versions reported by Eich et al. (9) and the previously validated Spanish version of the SCL-90-R.

Item	English	German	Spanish	Comment
2	Nervousness or shakiness inside	Nervosität oder innerem Zittern	Nerviosismo	Shortened but adequate.
9	Trouble remembering things	Gedächtnisschwierigkeiten	Tener dificultad para memorizar cosas	Equivalent.
11	Feeling easily annoyed or irritated	dem Gefühl, leicht reizbar oder verärgerbar zu sein	Sentirme enojado/a, malhumorado/a	Emotionally equivalent.
24	Temper outbursts you could not control	Gefühlsausbrüchen, denen sie gegenüber machtlos waren	Explotar y no poder controlarme	Very close.
28	Feeling blocked in getting things done	dem Gefühl, dass es Ihnen schwer fällt, etwas anzufangen	No poder terminar las cosas que empecé a hacer	Slight shift in focus (initiation vs. completion).
55	Trouble concentrating	Konzentrationsschwierigkeiten	Dificultades para concentrarme en lo que estoy haciendo	Aligned.
57	Feeling tense or keyed up	dem Gefühl aufgeregt oder gespannt zu sein	Sentirme muy nervioso/a, agitado/a	More intense.
74	Getting into frequent arguments	Der Neigung immer wieder in Auseinandersetzungen zu geraten	Meterme muy seguido en discusiones	Idiomatic and equivalent.
78	Feeling so restless you couldn’t sit still	So starker Ruhelosigkeit, dass sie nicht still sitzen können	Estar inquieto/a; no poder estar sentado/a sin moverme	More behavioural and specific.

No *de novo* translation or linguistic validation was conducted for the present study. The table is provided for transparency and to illustrate conceptual alignment across language versions. Minor lexical clarifications to the Spanish wording were introduced solely to enhance contextual clarity for Paraguayan Spanish speakers and do not constitute a formal translation or re-translation of the instrument.

## Appendix II Spanish adaptation of the SCL-ADHD subscale used in the present study.

**Table:** The following table presents the nine items of the Spanish version of the SCL-ADHD subscale employed in this study.

Item	Spanish version	Original reference (9)
2	Sentirme nervioso/a o con temblores por dentro	Nervousness or shakiness inside
9	Tener dificultad para memorizar cosas	Trouble remembering things
11	Sentirme enojado/a o malhumorado/a	Feeling easily annoyed or irritated
24	Explotar y no poder controlarme	Temper outbursts you could not control
28	No poder terminar las cosas que empecé a hacer	Feeling blocked in getting things done
55	Dificultades para concentrarme en lo que estoy haciendo	Trouble concentrating
57	Sentirme muy nervioso/a o agitado/a	Feeling tense or keyed up
74	Meterme muy seguido en discusiones	Getting into frequent arguments
78	Estar inquieto/a; no poder estar sentado/a sin moverme	Feeling so restless you couldn’t sit still

Items were rated on a five-point scale: 0 = not at all, 1 = a little bit, 2 = moderately, 3 = quite a bit, 4 = extremely. The wording reflects the final adaptation used in the Paraguayan correctional population, following the trilingual semantic review described in Appendix I. Response options were rated on a five-point Likert scale ranging from 0 (*not at all*) to 4 (*extremely*).
