# Supplementary material for: Screening-positive attention-deficit/hyperactivity disorder symptoms among incarcerated individuals in Paraguay: prevalence, psychological correlates, and criminological context
Source: Front Psychiatry. 2026 Apr 15;17:1752901. doi: 10.3389/fpsyt.2026.1752901 (PMC13125982; doi:10.3389/fpsyt.2026.1752901)
Supplement: Supplementary file 1 [file Table1.docx]

**Screening-Positive Attention-Deficit/Hyperactivity Disorder Symptoms among Incarcerated Individuals in Paraguay: Prevalence, Psychological Correlates, and Criminological Context**

**Supplementary Material**

**Correlations and collinearity diagnostics for SCL-90-R symptom dimensions**

The following supplementary tables provide detailed correlation matrices and variance inflation factor estimates to complement the sensitivity analyses reported in the main manuscript.

**Supplementary Table S1**

*Pearson correlations among SCL-90-R T-scores (N = 836)*

| **Variable pair** | **r (Pearson)** | **95% CI** | **p-value** | **N** |
| --- | --- | --- | --- | --- |
| DEP – ANS | 0.776 | 0.748 – 0.802 | < 0.001 | 836 |
| DEP – HOS | 0.586 | 0.540 – 0.629 | < 0.001 | 836 |
| DEP – OBS | 0.689 | 0.652 – 0.723 | < 0.001 | 836 |
| ANS – HOS | 0.648 | 0.607 – 0.686 | < 0.001 | 836 |
| ANS – OBS | 0.693 | 0.656 – 0.727 | < 0.001 | 836 |
| HOS – OBS | 0.574 | 0.527 – 0.618 | < 0.001 | 836 |

*Note.* DEP = Depression (T-score); ANS = Anxiety (T-score); HOS = Hostility (T-score); OBS = Obsessions–compulsions (T-score). All correlations are Pearson coefficients estimated in the full sample using continuous standardized T-scores. Confidence intervals were computed using Fisher’s z transformation.

**Supplementary Table S2**

*Partial correlations among SCL-90-R T-scores adjusted for sex (N = 836)*

| **Variable pair** | **Partial r (adjusted for sex)** | **95% CI** | **p-value** | **N** |
| --- | --- | --- | --- | --- |
| DEP – ANS | 0.783 | 0.755 – 0.808 | < 0.001 | 836 |
| DEP – HOS | 0.586 | 0.539 – 0.629 | < 0.001 | 836 |
| DEP – OBS | 0.712 | 0.677 – 0.744 | < 0.001 | 836 |
| ANS – HOS | 0.650 | 0.609 – 0.688 | < 0.001 | 836 |
| ANS – OBS | 0.696 | 0.659 – 0.729 | < 0.001 | 836 |
| HOS – OBS | 0.585 | 0.538 – 0.628 | < 0.001 | 836 |

*Note.* Partial correlations were estimated controlling for sex as a covariate (coded as a binary variable). All analyses were conducted using continuous standardized T-scores. Confidence intervals were derived using Fisher’s z transformation.

**Supplementary Table S3**

*Variance inflation factors (VIF) for multivariable modelling*

| **Variable** | **VIF** |
| --- | --- |
| DEP | 2.862 |
| ANS | 3.164 |
| HOS | 1.840 |
| OBS | 2.240 |

*Note.* VIF values below 5 are commonly considered indicative of absence of problematic multicollinearity. All predictors were entered simultaneously in the logistic regression model corresponding to the primary multivariable analysis.
